# The nut-and-bolt motion of a bacteriophage sliding along a bacterial flagellum: a complete hydrodynamics model

**DOI:** 10.1038/s41598-023-36186-1

**Published:** 2023-06-05

**Authors:** Sergey A. Karabasov, Mihail A. Zaitsev, Dmitry A. Nerukh

**Affiliations:** 1 School of Engineering and Materials Science, Queen Mary University of London, London, E1 4NS UK; 2 Nuclear Safety Institute, Moscow, Russia 115191; 3 Department of Mathematics, Aston University, Birmingham, B4 7ET UK

**Keywords:** Applied mathematics, Mechanical engineering, Computational biophysics

## Abstract

The ‘nut-and-bolt’ mechanism of a bacteriophage-bacteria flagellum translocation motion is modelled by numerically integrating the 3D Stokes equations using a Finite-Element Method (FEM). Following the works by Katsamba and Lauga (Phys Rev Fluids 4(1): 013101, 2019), two mechanical models of the flagellum-phage complex are considered. In the first model, the phage fiber wraps around the smooth flagellum surface separated by some distance. In the second model, the phage fiber is partly immersed in the flagellum volume via a helical groove imprinted in the flagellum and replicating the fiber shape. In both cases, the results of the Stokes solution for the translocation speed are compared with the Resistive Force Theory (RFT) solutions (obtained in Katsamba and Lauga Phys Rev Fluids 4(1): 013101, 2019) and the asymptotic theory in a limiting case. The previous RFT solutions of the same mechanical models of the flagellum-phage complex showed opposite trends for how the phage translocation speed depends on the phage tail length. The current work uses complete hydrodynamics solutions, which are free from the RFT assumptions to understand the divergence of the two mechanical models of the same biological system. A parametric investigation is performed by changing pertinent geometrical parameters of the flagellum-phage complex and computing the resulting phage translocation speed. The FEM solutions are compared with the RFT results using insights provided from the velocity field visualisation in the fluid domain.

## Introduction

Flagellotropic bacteriophages (phages) are viruses that infect bacteria using the bacterias’ flagella. The phage ‘wraps’ the flagellum with their tail (or head) fibers (‘legs’) and utilises flagellum’s rotation in such a way that the phage ‘screws in’ the flagellum like a nut on a bolt, eventually reaching the bacterial cell wall. This mechanism is being actively studied biologically and resulted in the discovery of many such phages^1–6^. The biological investigations are based on indirect conclusions drawn from various mutations of bacteria. For example, if a mutation leads to inability of flagellum rotation, the infection effectiveness by the phage is dramatically decreased. Similar effect results from the change of the direction of rotation. It turns out that counterclowise rotation is critically important. Since bacteria change the direction of flagellum’s rotation with the frequency of approximately 100 Hz, it has been concluded that at least one such period of rotation is enough for the phage to reach the bacterial wall.

One of the most studied flagellotropic phages is the so called χ-phage that infects E.coli and Salmonella. The phage’s philaments are attached to its tail and it uses them to attach to the flagellum (the ‘nut’). An alternative arrangement is used by the phage *φCbK* which has its filaments attached to its ‘head’^3^. In this case, when the phage reaches the bacterial cell wall, it uses its ‘tail’ to attach to the wall and inject its DNA in the bacteria.

To the best of our knowledge, there are no experimentally measured structures of the phage-flagella complex for any of the known flagellotropic phages. However, there are atomistic structures of the flagella themselves, such as *Salmonella’s* flagellum used by the χ-phage^1^ or *Caulobacter crescentus* flagellum used by the *φCbK* phage^3^.

The flagella consist of proteins ‘flagellins’ as well as polysaccharides ‘glycans’ and probably other biomolecules. For the purposes of this paper, an important point is that mechanically, both flagella show pronounced ‘grooves’ that can be used as the thread on the ‘bolt’. The presence of these grooves is decisive as the composition of the flagellum can be varied rather significantly, changing the atomistic structure of it, which does not preclude the phage from using its usual ‘nut and bolt’ mechanism of infecting the bacteria.

The molecular details of the structure of the flagella-phage complex as well as microbiologic studies provide evidence in favour of the mechanism. However, there are no direct observations of such motion. Therefore, mechanical modelling of the process, especially taking into account the hydrodynamics is very important for verifying the mechanism. Hydrodynamics is the key here because of the space and time scales of the process, which are well above the molecular microscopic scales usually considered when molecular structures of such biomolecular systems are investigated.

The first predictive model of the flagellum-phage complex was developed in^7^. This theoretical model focuses on the stationary translocation motion of the phage tail fibre along the rotating flaggelum, which altogther work as the mechanism of a nut screwing on a bolt discovered in^8^. The rapid transiton stage, when the phage first comes in contact with the bacterial flaggelum and wraps around it is neglected in the model. In general, the slow inertialess motion of the flagellum-phage complex in viscous liquid is described by the Stokes’ equations. However, instead of integrating the governing hydrodynamics equations, the Resistive Force Theory (RFT) of viscous hydrodynamics^9^ was used, which allows computing the drag forces and torques analytically. The simplificaton of the governing hydrodynamics equations is achieved by considering each part of the flagellum-phage complex as a slender body element in free space, whose motion is balanced by the local hydrodynamic drag described by some friction coefficients^10^.

In comparison with the direct integration of the Stokes Eqs. ^11^, the RFT neglects the long-range hydrodynamical interactions and evaluates the viscous forces exerted on the immersed body as a function of the local velocities only. While RFT has a low computational cost and it is simple to serve as a starting point for further analytical derivations^12^, the assumptions behind this model can be debated^13^. For example, several proposals exist for the best normal and tangential hydrodynamical friction coefficients, starting from the models proposed in the original works by Lighthill^14^ and Gray & Hancock^15^. However, as discussed in^16,17^, either choice of the RFT friction coefficients poorly captures the behaviour of helical flagella for the range of shapes of microswimmers present in nature. At the same time, the RFT models were shown to be able to produce accurate solutions, once their coeffcients are calibrated to match experimental observations^18^ or the results obtained by directly integrating the Stokes equations^19^.

In^7^, two RFT models of the flagellum-phage complex were conidered. In the first model, the relevant part of the bacterial flagellum was approximated by a smooth finite cylinder and a helicial fibre of the phage is placed at some distance from its surface. The phage tail is represented by another finite cylinder, and the phage head was represented by a sphere. Separate analytical friction coefficients were used for each element of the complex: the two finite cylinders, the helical fibre, and the sphere. In the second model, instead of a smooth cylinder, the bacterial flagellum was represented by a cylinder with a helical groove, partly filled by the fibre. In this case, the helical fibre was guided by the groove in its helical motion, and its friction coefficient was computed assuming a fully developed shear flow resisting the sliding between the fiber and the groove surface. It was shown that both mechanical models of the flagellum-phage complex correctly predicted the general property of the mechanical ‘nut-and-bolt’ system: the connection between the fibre chirality, the direction of rotation of the flagellum, and the direction of the fiber’s translocation as well as predicting the translocation speed in the order of 1 μm/s in accordance with the experimental observations^8^. However, in comparison with the first model, the results of the second model predicted the opposite trend for the translocation velocity variation with changing the length of the phage tail. The qualitative difference between the two RFT models was explained by the fact that the second model includes grooves, similar to the ones observed experimentally^1^, hence, it is arguably more physically refined. However, it remains unclear how well the RFT approximation is justified for computing the flow about the fibre sliding around the cylindrical surface and the fibre sliding in the helical groove to represent the hydrodynamics of the flagellum-phage interaction. Hence, the goal of the current work is to implement the same two mechanical models of the flagellum-phage without resorting to RFT approximtions. Following the approach of^19^, the drag forces and torques will be computed numerically by integrating the governing Stokes equations with taking into account boundary conditions for the complete 3D surface geometry and including non-local hydrodynamic interactions.

The paper is organised as follows. In the Methods section, following^7^, two mechanical models are introduced to describe the locomotion of the flagellum-phage complex in inertialess viscous fluid. The governing equations of the suitable linearised approach to compute the total forces and torques of the system are considered for the two models of the flagellum-phage complex. The numerical approach for solving the associated Stokes boundary value problems using a finite-element method is desrcribed. The results of the numerical calcultions are presented and compared with the RFT theory for a range of pertinent geometrical parameters of the flagellum-phage complex in the Results section, followed by Conclusions.

## Methods

### Mechanical models of the flagellum-phage complex

**Figure 1:**
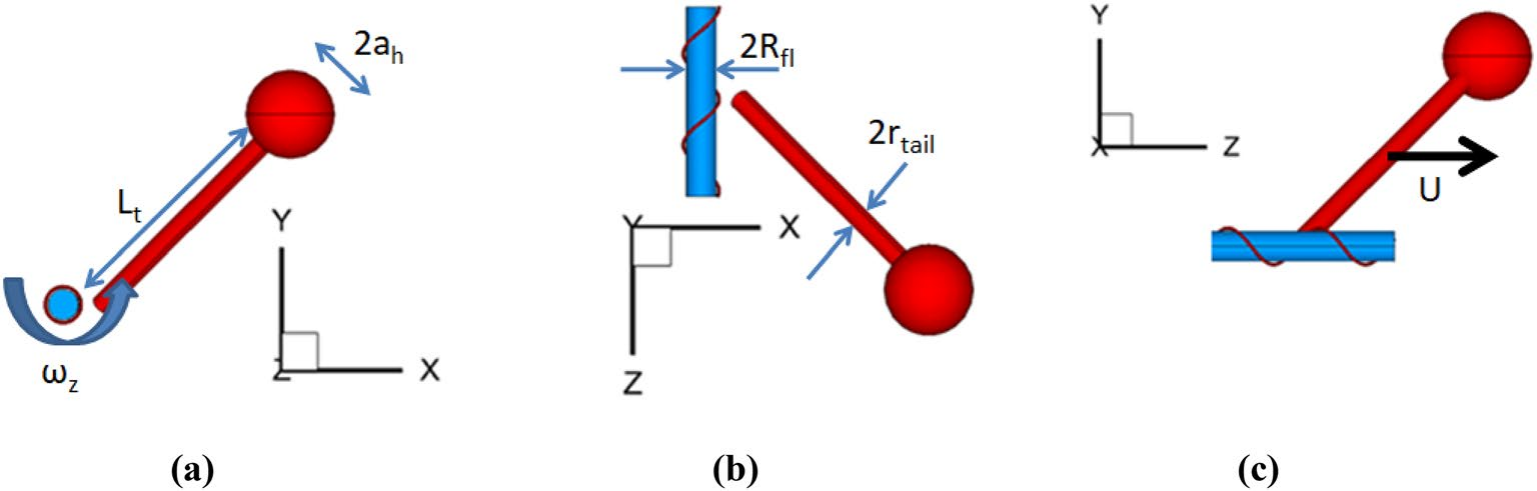
3D views of the flagellum-phage model in *x–y* plane (**a**), *z-x* plane (**b**), and *y–z* plane (**c**). The flagellum rotates in the x–y plane so that *ω*_*fl*_ = *ω*_*z*_ .

Following^7^, the flagellum-phage complex is represented by a mechanical model, where the phage consists of a spherical head of radus *a*_*h*_, a cylindrial tail of length *L*_*t*_ and radius *r*_*tail*_, a helical fiber of length *L*_*fib*_ and radius *r*_*fib*_ with the helix angle *α* = 51°. The phage tail is positioned at a 45^o^ angle relative to the flagellum centreline which is aligned with the z-axis (Fig. 1). The bacterial flagellum is represented by a finite cylinder of radius *R*_*fl*_. In reality, the flagellum length is much longer than the lateral size of the flagellum-phage complex. Hence, two different flagellum lengths were tested to ensure the numerical modelling results are virtually independent of the conditions on the open ends of the flagellum cylinder so long as the phage geometry is fully included. The flagellum rotates around the centreline at frequency 100 Hz *, ω*_*fl*_ = 2π*f*, *f* = 100 Hz, and the surrounding water is considered at standard conditions.

In model 1, the flagellum cylinder is assumed to be smooth and the distance between the flagellum surface and the centre line of the fibre is equal to *d* = 2*r*_*fib*_. In accordance with this model, the coordinates of the central line of the fibre are described by

1 $$ {\varvec{r}}_{fib} \left( l \right) = \left[ {\left( {R_{fl} + d} \right){\text{cos}}\left( {\frac{l}{{{\raise0.7ex\hbox{${\left( {R_{fl} + d} \right)}$} \!\mathord{\left/ {\vphantom {{\left( {R_{fl} + d} \right)} {{\text{sin}}\alpha }}}\right.\kern-0pt} \!\lower0.7ex\hbox{${{\text{sin}}\alpha }$}}}}} \right),h\left( {R_{fl} + d} \right){\text{sin}}\left( {\frac{l}{{{\raise0.7ex\hbox{${\left( {R_{fl} + d} \right)}$} \!\mathord{\left/ {\vphantom {{\left( {R_{fl} + d} \right)} {{\text{sin}}\alpha }}}\right.\kern-0pt} \!\lower0.7ex\hbox{${{\text{sin}}\alpha }$}}}}} \right),z_{0} + l{\text{cos}}\alpha } \right],\;0 \le l \le L_{fib} , $$

where *z*_0_ is the coordinate of the beginning of the fibre, the chirality index *h* is equal to 1 for the clockwise helix and −1 for the counter-clockwise helix.

In model 2, a helical groove is cut in the cylindrical surface of the flagellum, which is partly filled by the fibre so that the gap between the fibre and the bottom of the groove is *h*_*gap*_ = *r*_*fib*_/2, and a half of the fibre circumference is immersed in the groove. In this case the coordinates of the central line of the fibre are given by

2 $$ {\varvec{r}}_{fib} \left( l \right) = \left[ {R_{fl} {\text{cos}}\left( {\frac{l}{{{\raise0.7ex\hbox{${R_{fl} }$} \!\mathord{\left/ {\vphantom {{R_{fl} } {{\text{sin}}\alpha }}}\right.\kern-0pt} \!\lower0.7ex\hbox{${{\text{sin}}\alpha }$}}}}} \right),hR_{fl} {\text{sin}}\left( {\frac{l}{{{\raise0.7ex\hbox{${R_{fl} }$} \!\mathord{\left/ {\vphantom {{R_{fl} } {{\text{sin}}\alpha }}}\right.\kern-0pt} \!\lower0.7ex\hbox{${{\text{sin}}\alpha }$}}}}} \right),z_{0} + l{\text{cos}}\alpha } \right],\;0 \le l \le L_{fib} ,{ } $$

where all notations are the same as in Eq. (1) for model 1. In the model 2 case the centre line lies on the bacterial flagellum surface and a part of the fibre is immered inside the helical groove.

In each model, in response to the flagellum motion, the phage rotates around the flagellum at frequency ω_p_ and acquires a translocation velocity *U* along the flagellum centreline. Both of these parameters can be found from the Stokesian solution of the flagellum-phage interaction problem, as discussed in the following section.

Further geometrical parameters are defned in accordance with^20,21^ and^7^ and summarised in Table 1.

**Table 1:** Geometrical parameters of elements of the flagellum-phage models.

Element	Length, nm	Radius, nm
Bacterial flagellum	124–248	10
Phage head	–	30
Phage tail	220	7
phage fibre	200	1

**Figure 2:**
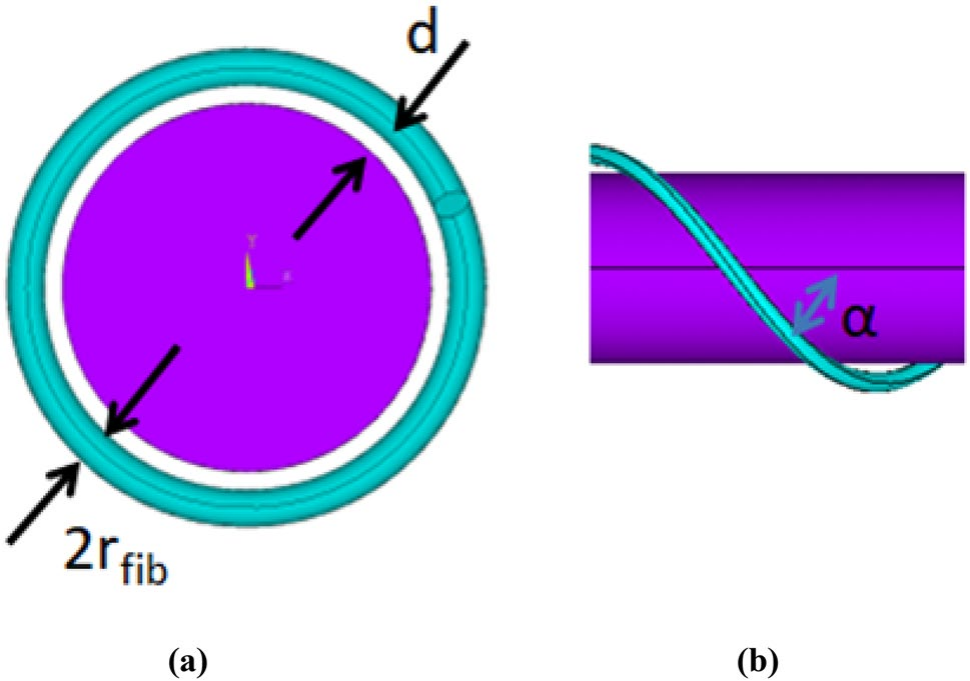
Model 1 of the fibre helix around a smooth cylindrical surface of the flagellum: front view (**a**) and lateral view (**b**).

**Figure 3:**
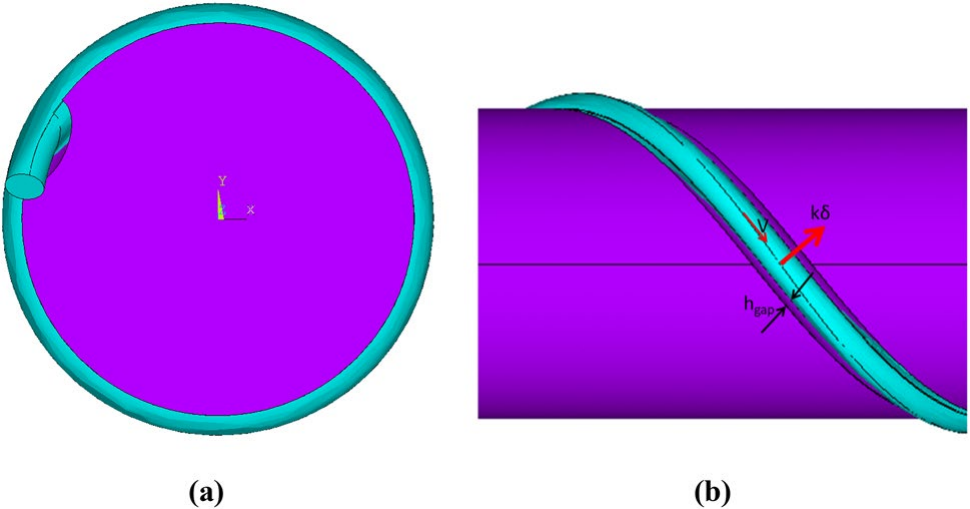
Model 2 of the fibre helix partly filling the groove of the cylindrical flagellum: front view (**a**) and lateral view (**b**).

Figures 2 and 3 show zoomed-in views of the flagellum-fibre geometry for models 1 and 2, respectively. In comparison with model 1, the fibre in model 2 is closely immersed in the groove, which leads to their restoring interaction. Following^7^, the interaction is modelled by a repelling force per unit fibre length, arising from a restoring potential 0.5 *kδ*^2^ , where *δ* is the distance from the centre of the potential well and k is a constant. The force is exerted normally to the fibre centre line and the cylinder surface. The magnitude of the reaction force per unit length *kδ* is determined from the force balance (see the following section). In comparison with model 1 the fibre motion in model 2 rigidly follows the groove helix so that its absolute velocity *V* is aligned with the local tangent to the helix centre line.

**Figure 4:**
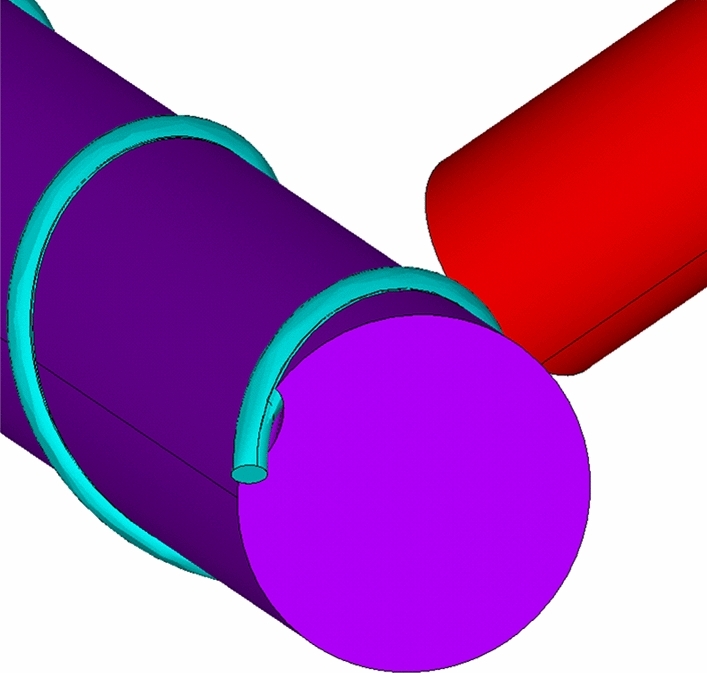
A close-up view of the flagellum-phage complex in the vicinity of the phage tail and the fibre of model 2.

Since 3D geometry details describing how the cylindrical flagellum surface is merged into the helical fibre ligament of the phage are not available in^7^, a small gap is introduced between the flagellum cylinder and the fibre helix for simplicity. To illustrate this, Fig. 4 shows a close-up view of the gap between a part of the page tail and the flagellum with the fibre for model 2.

### Fluid–structure interaction modelling

Following^11^, the total force in the flagellum-phage system can be computed by integrating the stress tensor ${T}_{ij}$, where *i*,*j* = *x*,*y*,*z* are Cartesian coordinates, multiplied by the local area normal vector $d{s}_{i}$ over the phage surface. In the same manner, the total torque of the system is equal to the surface integral of a vector product of the stress tensor with a radius vector of the surface multiplied by the local area normal. Both the total force and the total torque are zero for the steady motion. In particular, the z-axis components of the force and the torque are zero too, given by

3 $$ F_{z} = \iint {T_{iz} ds_{i} = 0\quad {\text{and}}\quad M_{z} } = \iint {\left( {xT_{iy} - yT_{ix} } \right)ds_{i} = 0,}\;{\text{i}} = {\text{x}},{\text{y}},{\text{z}}, $$

where the integrals are evaluated over the phage surface and summation over a repeated index is assumed. In Model 2 additional restoring nut-bolt forces are considered.

Models 1 and 2 include three degrees of freedom each, so that ${F}_{z}={F}_{z}\left({q}_{1},{q}_{2},{q}_{3}\right)$ and ${M}_{z}={M}_{z}({q}_{1},{q}_{2},{q}_{3})$. In the case of model 1, the degrees of freedom ${q}_{1},{q}_{2},$ and ${q}_{3}$ are: (1) the rotation frequency of the flagellum ω_fl_, (2) the rotation frequency of the phage,ω_p_, and (3) the translocation velocity of the phage U, respectvely. In the case of model 2, where the phage and flagellum rotations are tightly coupled, these are: (1) the rotation frequency of the flagellum ω_fl_, (2) the displacement of the fibre δ, and (3) the velocity of the fibre helix *V* .

Following^7^ and assuming a linear relationship between the force and the torque and the degrees of freedom, a linearised version of Eq. (3) is written by treating the values of the degrees of freedom *q*_*i*_ as perturbations about the equilibrium state corresponding to the system at rest:

4 $$ \left( {\frac{{\partial F_{z} }}{{\partial q_{i} }}} \right)_{{{\varvec{q}} = 0}} q_{i} = 0\quad {\text{and}}\quad \left( {\frac{{\partial M_{z} }}{{\partial q_{i} }}} \right)_{{{\varvec{q}} = 0}} q_{i} = 0,\quad i = 1,2,3 $$

(note the summation over a repeated index *i* again).

For a slow motion of the flagellum-phage complex, $\varepsilon$, considering unit perturbations of the degrees of freedom one at a time, the partial derivatives in Eq. (4) are approximated using L'Hôpital’s rule:

5 $$ \begin{gathered} \frac{{\partial F_{z} }}{{\partial q_{1} }} = \frac{{F_{z} \left( {\varepsilon ,0,0} \right)}}{\varepsilon },\frac{{\partial F_{z} }}{{\partial q_{2} }} = \frac{{F_{z} \left( {0,\varepsilon ,0} \right)}}{\varepsilon },\frac{{\partial F_{z} }}{{\partial q_{3} }} = \frac{{F_{z} \left( {0,0,\varepsilon } \right)}}{\varepsilon }\;{\text{and}} \hfill \\ \frac{{\partial M_{z} }}{{\partial q_{1} }} = \frac{{M_{z} \left( {\varepsilon ,0,0} \right)}}{\varepsilon },\frac{{\partial M_{z} }}{{\partial q_{2} }} = \frac{{M_{z} \left( {0,\varepsilon ,0} \right)}}{\varepsilon },\frac{{\partial M_{z} }}{{\partial q_{3} }} = \frac{{M_{z} \left( {0,0,\varepsilon } \right)}}{\varepsilon }. \hfill \\ \end{gathered} $$

For model 1, substituting (5) into (4) leads to the following system of equations for the linear force and torque coefficients

6 $$ C_{1} \omega_{fl} + C_{2} \omega_{p} + C_{3} U = 0\quad {\text{and}}\quad C_{4} \omega_{fl} + C_{5} \omega_{p} + C_{6} U = 0, $$

where

7 $$ C_{1} = \frac{{F_{z} \left( {\varepsilon ,0,0} \right)}}{\varepsilon },\;C_{2} = \frac{{F_{z} \left( {0,\varepsilon ,0} \right)}}{\varepsilon },\;C_{3} = \frac{{F_{z} \left( {0,0,\varepsilon } \right)}}{\varepsilon },\;C_{4} = \frac{{M_{z} \left( {\varepsilon ,0,0} \right)}}{\varepsilon },\;C_{5} = \frac{{M_{z} \left( {0,\varepsilon ,0} \right)}}{\varepsilon },\;{\text{and}}\;C_{6} = \frac{{M_{z} \left( {0,0,\varepsilon } \right)}}{\varepsilon }. $$

In the above equations, ${F}_{z}(\varepsilon ,\mathrm{0,0})$ and ${M}_{z}(\varepsilon ,\mathrm{0,0})$ correspond to the z-components of the drag force and its torque acting on the phage during a slow rotation of the flagellum at ${\omega }_{fl}=\varepsilon$ while the phage is at rest, ${F}_{z}(0,\varepsilon ,0)$ and ${M}_{z}(0,\varepsilon ,0)$ correspond to the z-components of the drag force and its torque acting on the phage during its slow rotation at ${\omega }_{p}=\varepsilon$ while the flagellum is at rest, and ${F}_{z}(\mathrm{0,0},\varepsilon ,)$ and ${M}_{z}(\mathrm{0,0},\varepsilon )$ correspond to the z-components of the drag force and its torque acting on the phage during its slow translation along the flagellum at $U=\varepsilon$ while the flagellum is at rest.

For model 2, following^7^ and defining the local binormal vector to the fibre centre line

8 $$ {\varvec{b}}_{fib} \left( l \right) = \left[ {{\text{cos}}\alpha {\text{sin}}\left( {\frac{l}{{{\raise0.7ex\hbox{${R_{fl} }$} \!\mathord{\left/ {\vphantom {{R_{fl} } {{\text{sin}}\alpha }}}\right.\kern-0pt} \!\lower0.7ex\hbox{${{\text{sin}}\alpha }$}}}}} \right),{\text{cos}}\alpha {\text{cos}}\left( {\frac{l}{{{\raise0.7ex\hbox{${R_{fl} }$} \!\mathord{\left/ {\vphantom {{R_{fl} } {{\text{sin}}\alpha }}}\right.\kern-0pt} \!\lower0.7ex\hbox{${{\text{sin}}\alpha }$}}}}} \right),h{\text{sin}}\alpha } \right],\;0 \le l \le L_{fib} , $$

the local restoring force per unit length $h\cdot k\delta {{\varvec{b}}}_{fib}$ and its torque are analytically intgrated over the fibre length and the following system of equations is obtained:

9 $$ C_{1} \omega_{fl} + hL_{fib} k\delta sin\alpha + C_{3} V = 0\quad {\text{and}}\quad C_{4} \omega_{fl} - hL_{fib} R_{fib} k\delta cos\alpha + C_{6} V = 0. $$

Here ${C}_{1}$, ${C}_{3}$, ${C}_{4}$, and ${C}_{6}$ are defined in accordance with (7), where ${F}_{z}(\varepsilon ,\mathrm{0,0})$ and ${M}_{z}(\varepsilon ,\mathrm{0,0})$ correspond to the z-components of the drag force and its torque acting on the phage during its slow rotation together with the flagellum at ${\omega }_{fl}=\varepsilon$ and ${F}_{z}(\mathrm{0,0},\varepsilon ,)$ and ${M}_{z}(\mathrm{0,0},\varepsilon )$ correspond to the z-components of the drag force and its torque acting on the phage during its cock-crew-like motion following the flagellum groove at velocity ${\varvec{V}}=\left(-\varepsilon y\frac{sin\alpha }{{R}_{fl}},\varepsilon x\frac{sin\alpha }{{R}_{fl}},\varepsilon cos\alpha \right)$, where ${\text{V = }}\left\lfloor {\mathbf{V}} \right\rfloor$ and the flagellum is at rest.

To solve Eqs. (6) for the translocation velocity U, six coefficients ${C}_{1}$ $-$ ${C}_{6}$ must be defined. For Eqs. (9), four coefficients ${C}_{1}$, ${C}_{3}$, ${C}_{4}$, and ${C}_{6}$ are still needed in order to compute $V$ from which the translocation velocity can be obtained as $U = V\cos \alpha .$

Similar governing equations to (6) and (9) were obtained in^7^. However, in the latter work, the Resistivity Force Theory (RFT) was invoked to define the unknown force and momentum coefficients by first representing the drag forces and torques on the phage as a linear combination of local forces and torques of its separate elements (fibre, tail, head) and then using analytical models to compute the friction coefficients for each geometrical element. In the current investigation the Stokes equations are numerically solved to obtain the forces and torques corresponding to each elementary slow motion of the flagellum-phage system, thereby directly obtaining the force and momentum coefficients using the definition (7), without any RFT assumptions.

### Governing flow equations and numerical solution approach

**Figure 5:**
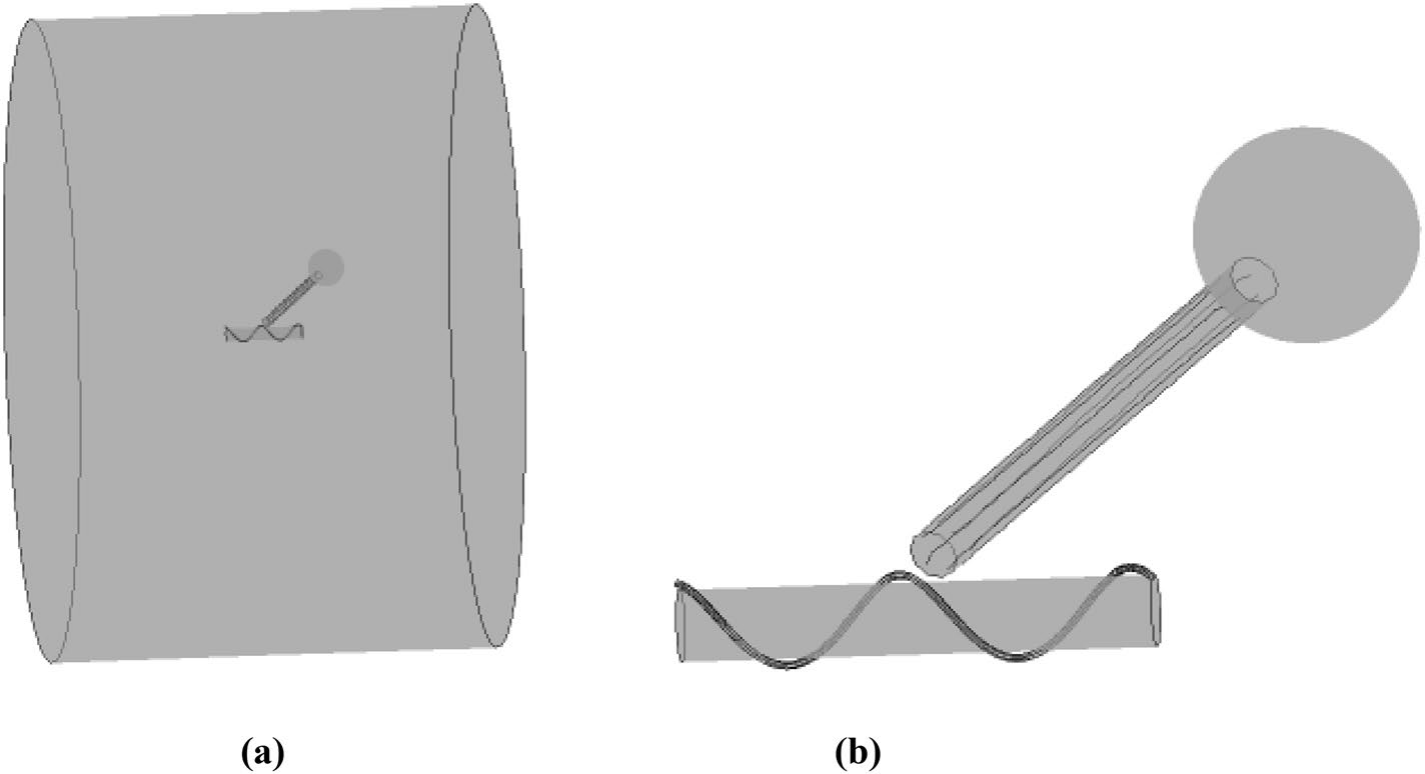
Computational domain for numerical solution of the Stokes boundary value problem for the flagellum—phage complex: full domain (**a**) and close-up view of the flagellum—phage geometry (**b**).

For each of the 3 elementary motions of model (6) and 2 elementary motions of model (9) the governing Stokes equations,

10 $$ \begin{aligned} \nabla \cdot {\varvec{v}} & = 0, \\ \nabla {\text{p}} & = \mu {\Delta }{\varvec{v}}, \\ \end{aligned} $$

where ${\varvec{v}}={(u,v,w)}^{T}$ is the Cartesian velocity vector of water flow around the flagellum-phage complex, *μ* is a dynamic viscosity coefficient, and *p* is hydrostatic pressure, are considered. Appropriate non-slip boundary conditions are imposed on the moving and stationary parts of the flagellum-phage complex in accordance with the definitions of the elementary motions in (7). Zero velocity is imposed on all external domain boundaries, sufficiently far away from the flagellum-phage complex to neglect the numerical open-boundary condition effect. The boundary-value problem is solved in the computational domain shown in Fig. 5.

The governing partial differential problem was solved with the Finite Element Method (FEM), which was previously validated in simulations of spermatozoon locomotion in^13,19^. Details of the FEM method are summarised below. Following the standard approach^22^, the FEM with second-order base functions is implemented in the framework of the penalty method, which requires minimisation of the functional as follows:

11 $$ J\left( {u,v,w} \right) = \lambda \mathop \smallint \limits_{V} \left( \Delta \right)^{2} dV + 2\mu \mathop \smallint \limits_{V} \left( {\varepsilon_{xx}^{2} + \varepsilon_{yy}^{2} + \varepsilon_{zz}^{2} + \frac{1}{2}\varepsilon_{xy}^{2} + \frac{1}{2}\varepsilon_{xz}^{2} + \frac{1}{2}\varepsilon_{yz}^{2} } \right)dV - \mathop \smallint \limits_{V} \left( {f_{x} u + f_{y} v + f_{z} w} \right)dV $$

with penalty a parameter λ, where ${\varepsilon }_{xx},{\varepsilon }_{yy},{\varepsilon }_{zz},{\varepsilon }_{xy},{\varepsilon }_{xz},{\varepsilon }_{yz}$ are the components of the strain rate tensor

$$ \left( {\Delta ,\varepsilon_{xx} ,\varepsilon_{yy} ,\varepsilon_{zz} ,\varepsilon_{xy} ,\varepsilon_{xz} ,\varepsilon_{yz} } \right) = \left( {\frac{\partial u}{{\partial x}} + \frac{\partial v}{{\partial y}} + \frac{\partial w}{{\partial z}},\frac{\partial u}{{\partial x}},\frac{\partial v}{{\partial y}},\frac{\partial w}{{\partial z}},\frac{1}{2}\left( {\frac{\partial u}{{\partial y}} + \frac{\partial v}{{\partial x}}} \right),\frac{1}{2}\left( {\frac{\partial u}{{\partial z}} + \frac{\partial w}{{\partial x}}} \right),\frac{1}{2}\left( {\frac{\partial v}{{\partial z}} + \frac{\partial w}{{\partial y}}} \right)} \right) $$

and ${f}_{x},{f}_{y},{f}_{z}$ are internal forces. The discretisation results in a sparse system of linear algebraic equations that is solved using a direct method based on lower–upper decomposition^23^. The Intel Math Kernel Library solver is used for solving the linear system of equations.

**Figure 6:**
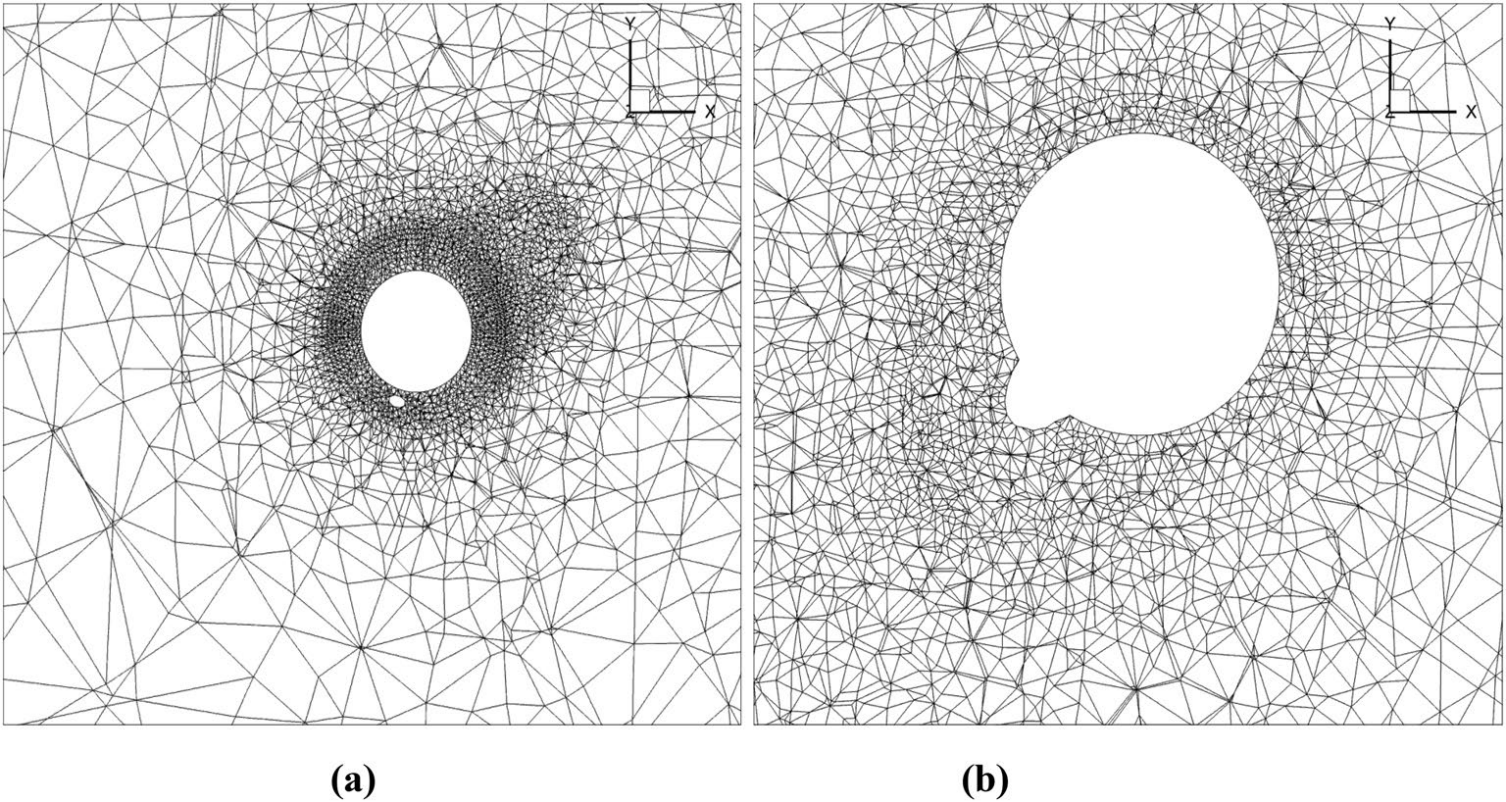
A slice of the computational grid for model 1 including the flagellum and the fibre in a plane normal to the flagellum axis (**a**) and a zoomed-in view around the phage head (**b**).

**Figure 7:**
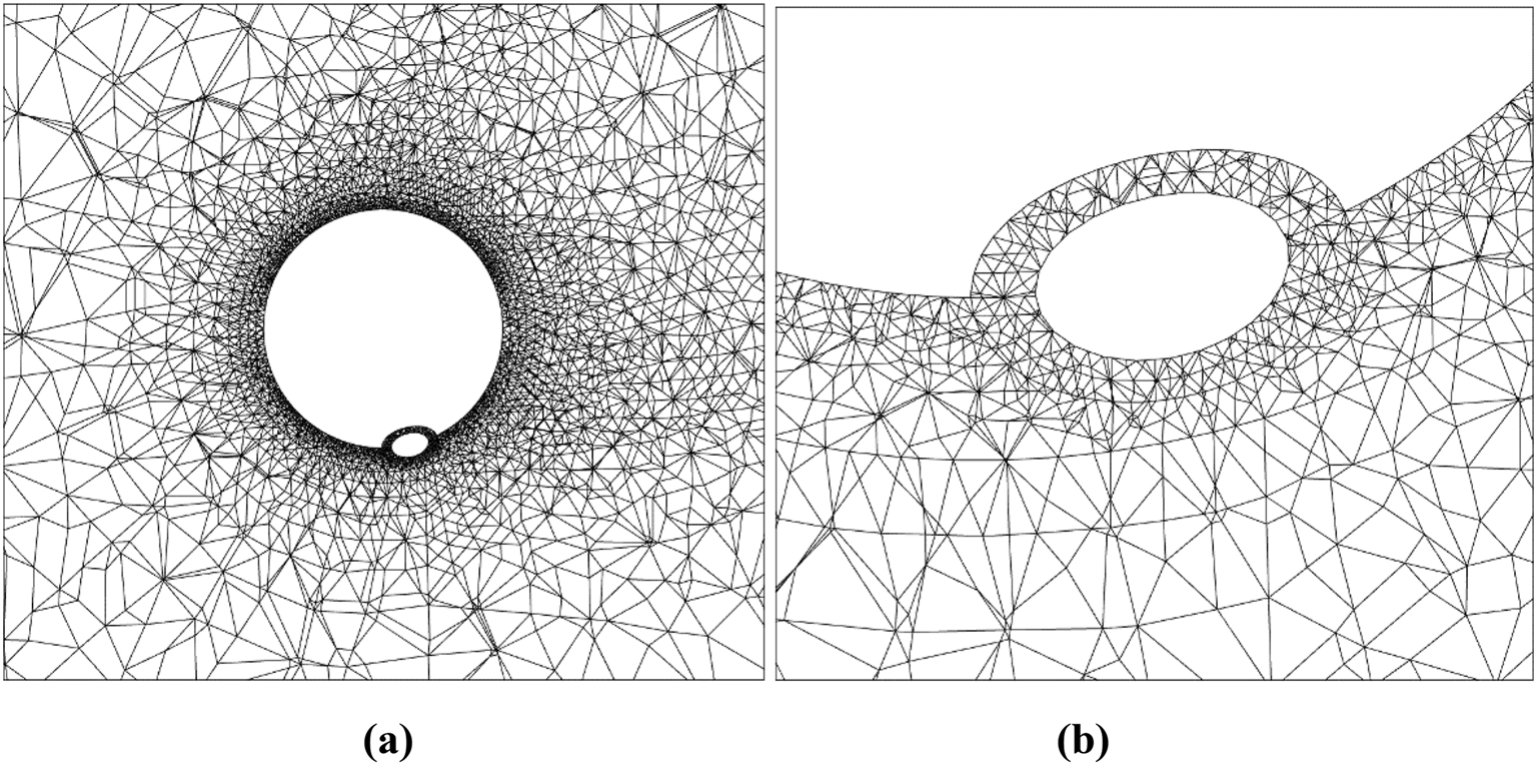
A slice of the computational grid for model 2 including the flagellum and the fibre in a plane normal to the flagellum axis (**a**) and a zoomed-in view (**b**).

For FEM solution the computational domain was discretised by an unstructured grid. Figure 6 shows the computational grid details in an *x*–*y* cross-section of the flagellum-phage flow for model 1, and Fig. 7 shows the same for model 2. It can be noted that the FEM grid is locally refined near the solid surfaces to capture the flow gradients. Additional computational grid details are summarised in Table 2. Numerical grid sensitivity calculations showed that the forces and momentum computed are virtually independent on further grid refinement. Notably, the moderate grid enabled performing all calculations on a workstation computer.

**Table 2:** Numerical grid resolution for solving the Stokes problem.

Element	Phage fibre	Phage tail and head	Spherical domain around the phage head and the flagellum	Rest of the domain
Grid cell size, nm	0.75	2.5	25	50

## Results

**Figure 8:**
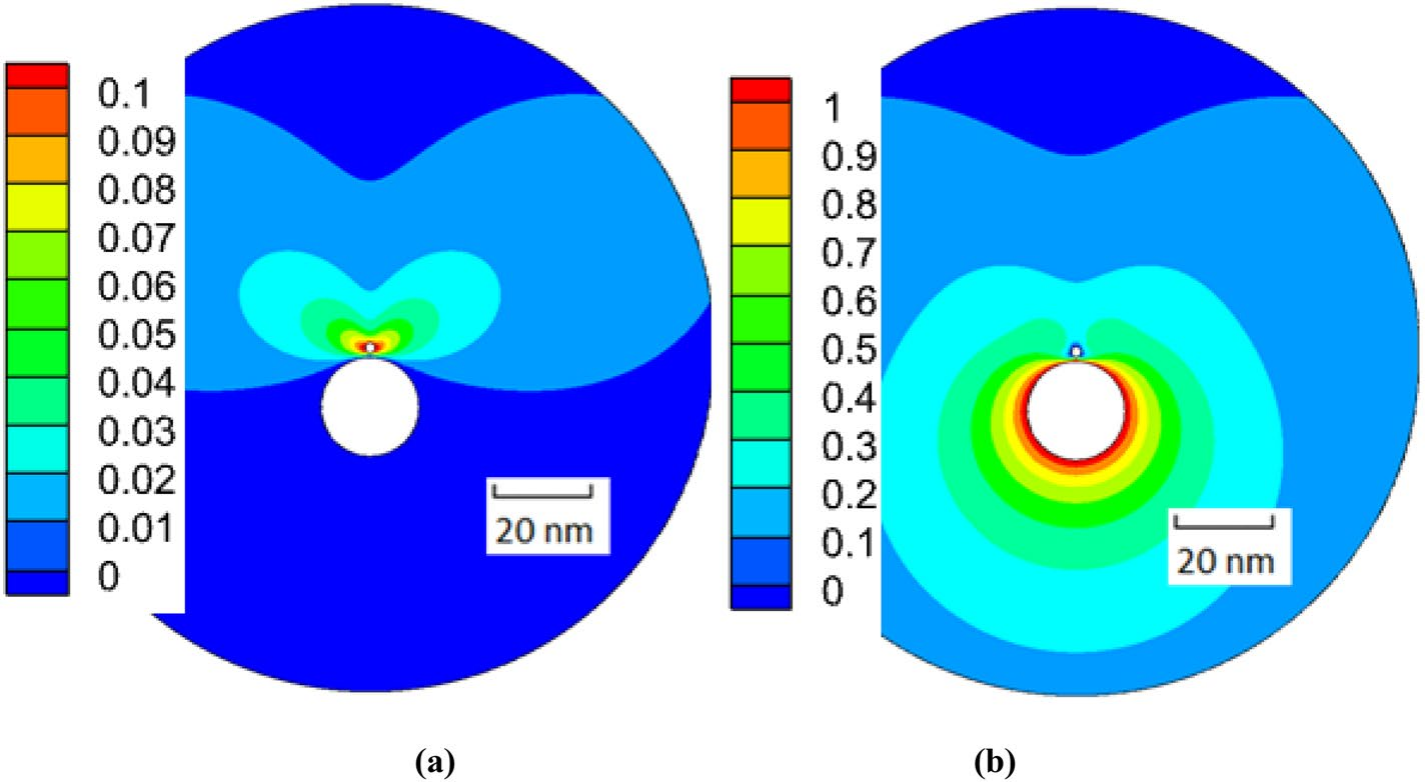
Distributions of the velocity magnitude in the x–y plane of the flagellum-phage complex of model 1 for the elementary motions of fibre translocation (**a**) and flagellum rotation (**b**). Dimensionless units of velocity are used based on 6.28 μm/s.

Figure 8 shows the distribution of the velocity magnitude in the plane normal to the flagellum cylinder for the elementary motions of model 1 using (6) and (7). Panel (a) shows the velocity distribution around the translocating fiber corresponding to ${C}_{3}$ and ${C}_{6}$ coefficients, panel (b) shows the results for the flagellum rotation from which the coefficients ${C}_{1}$ and ${C}_{4}$ are obtained. In comparsion wth these, the translocaton and rotation motions of model 2 are rigidly coupled in accordance with the helical groove geometry.

**Figure 9:**
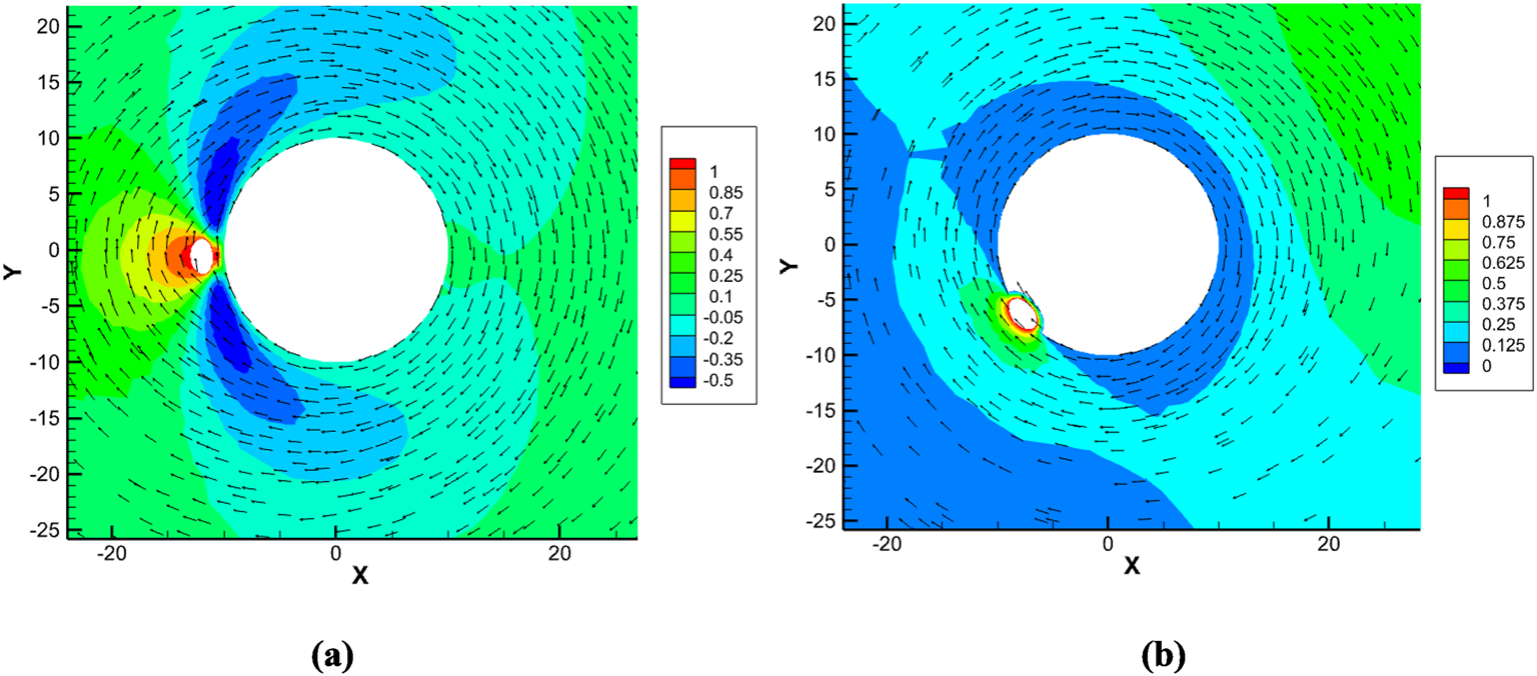
A cross-plane view on the flow velocity details in the vicinity of the phage-flagellum complex for model 1 (**a**) and 2 (**b**). The colormap shows the z-velocity component normalised by the translocation speed in each case, and the flow directionality in the (x–y) plane is indicated by arrows. The length units in the x- and y-axis are in nm.

Figure 9 compares velocty field distributions zooming in the phage-flagellum complex for the Stokes flow models 1 and 2 in a cross-plane normal to the flagellum centreline. The cross-plane is located at a half distance between the phage tail and the flagellum edge. For each model the helix direction corresponds to a positive translocation speed with respect to the z-axis (Fig. 1). Notably, the translocation of the fibers suspended above the flagelar surface (Fig. 9a) generates wake flows, which drag the fluid in the opposite direction compared to the phage motion. The effect of distributed wake flow regions on the phage motion would be challenging to account for in the RFT model. In comparison with this, in model 2, half of the fibre circumference is immersed in the groove and the effect of the fibre motion on the surrounding flow is much smoother, without any noticeable wakes. Hence, the groove provides a mechanism for the phage to reduce the kinetic energy losses associated with perturbing the viscous flow around the flagellum.

**Figure 10:**
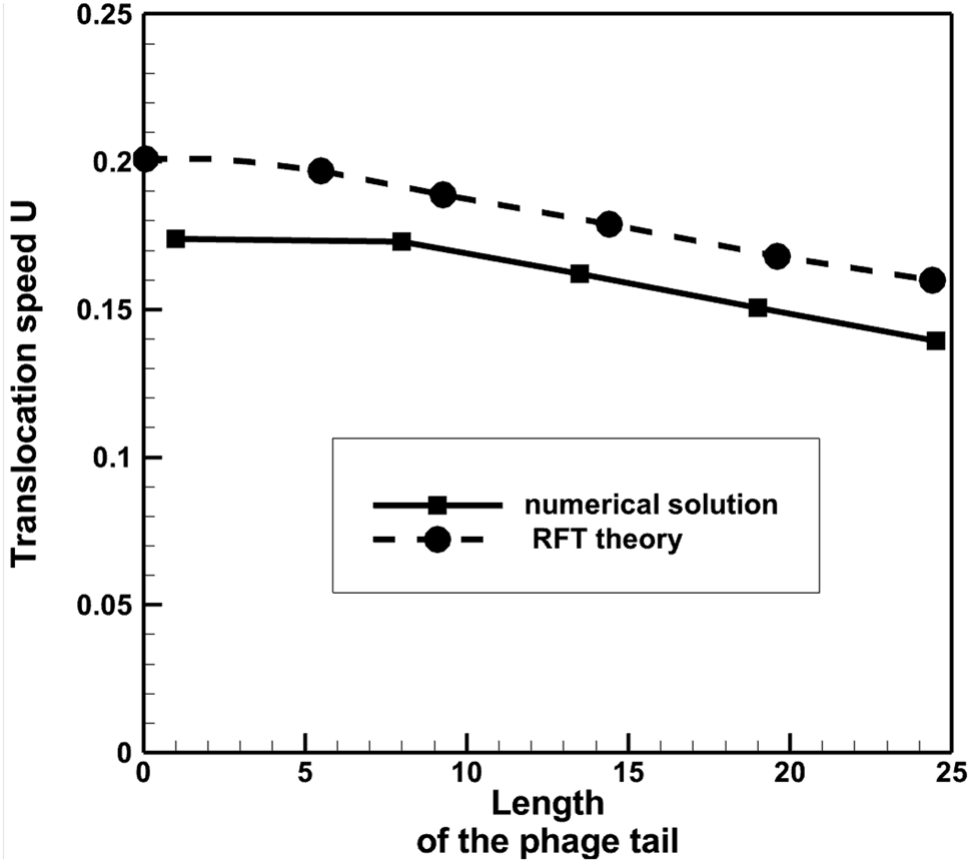
Dependency of the phage translocation speed on its tail length: the Stokes solution vs. the RFT theory with the recommended value of the perpendicular friction coefficient ${\zeta }_{\perp ,fib}$ from^7^ for model 1. Dimensionless units of velocity and length are used based on 6.28 μm/s and 10 nm, respectively.

The Stokes equations of model 1 were solved for a range of phage tail lengths to compare with the results of the RFT simulations in^7^. Figure 10 shows the simulation results, where the same non-dimensionalisation of the phage translocation speed and tail lenth was used as in^7^ based on the flagellum radius and rotation frequency, *R*_*fl*_ and ${\omega }_{fl}$. Both the Stokes and the RFT solutions^7^ of model 1 show a decay of the translocation speed with increasing the phage tail length. At the same time, the FEM solution consistently predicts a 15–20% lower value of the translocation speed compared to the RFT theory. This difference is much larger than the uncertanty of the FEM modelling associated with finite grid resolution and, therefore, is attributed to approxmations of the RFT model. In particular, following^7,24^, analytical approximations for friction coefficients were used, i.e. the proportionality coefficients between the local drag force and the velocity components perpendicular and parallel to the local tangent of the fibre,

12 $$ \zeta_{ \bot ,fib} = \frac{4\pi \mu }{{Ln\left( {2d/r_{fib} } \right)}}\quad {\text{and}}\quad \zeta_{\parallel ,fib} = \frac{1}{2}\zeta_{ \bot ,fib} , $$

which are based on applying a symmetry boundary condition to represent the interaction of a cylindrical fibre rod with a locally planar flagellar surface, thereby ignoring 3D curvature effects.

**Figure 11:**
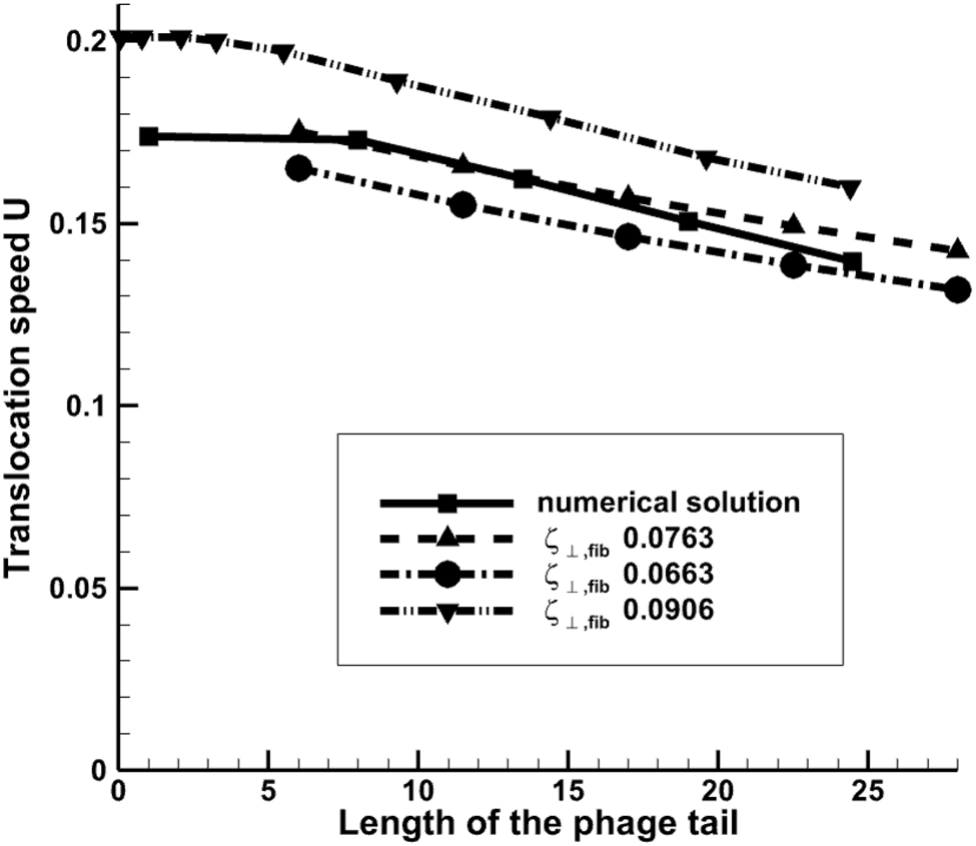
Dependency of the phage translocation speed on its tail length: the Stokes solution vs. the RFT theory with several values of the perpendicular friction coefficient $\zeta_{ \bot ,fib}$ for model 1. Dimensionless units of velocity and length are used based on 6.28 μm/s and 10 nm, respectively. ${\zeta }_{\perp ,fib}=0.00906$ correspond to the value from^7^. Other values are directly obtained from the Stokes solution.

To assess the effect of the error of the perpendicular friction coefficient $\zeta_{ \bot ,fib}$ the same coefficient has been calculated directly from the Stokes flow solution. In comparison with the friction coefficient value obtained from Eq. (12), ${\zeta }_{\perp ,fib}=0.00906$, the Stokes calculation corresponding to the elementary translocation motion of the fiber (${C}_{3}$ and ${C}_{6}$ coefficients in (6)) leads to ${\zeta }_{\perp ,fib}=0.00763$. Interestingly, the Stokes calculation using the elementary motion corresponding to the flagellum rotation (coefficients ${C}_{1}$ and ${C}_{4}$ in (6)) results in a slightly smaller value, ${\zeta }_{\perp ,fib}=0.00663$. Both numerical values of the friction coefficient are subsituted in the RFT model of^7^, which was implemented for the sake of comparison with the complete Stokes solution. Figure 11 compares the results of all three RFT solutions of model 1, which are only different by the ${\zeta }_{\perp ,fib}$ value with the Stokes solution of the same model. It can be noted that the RFT model with the numerical friction coefficient corresponding to the elementary motion of fibre translocation agrees with the Stokes solution particularly well.

**Figure 12:**
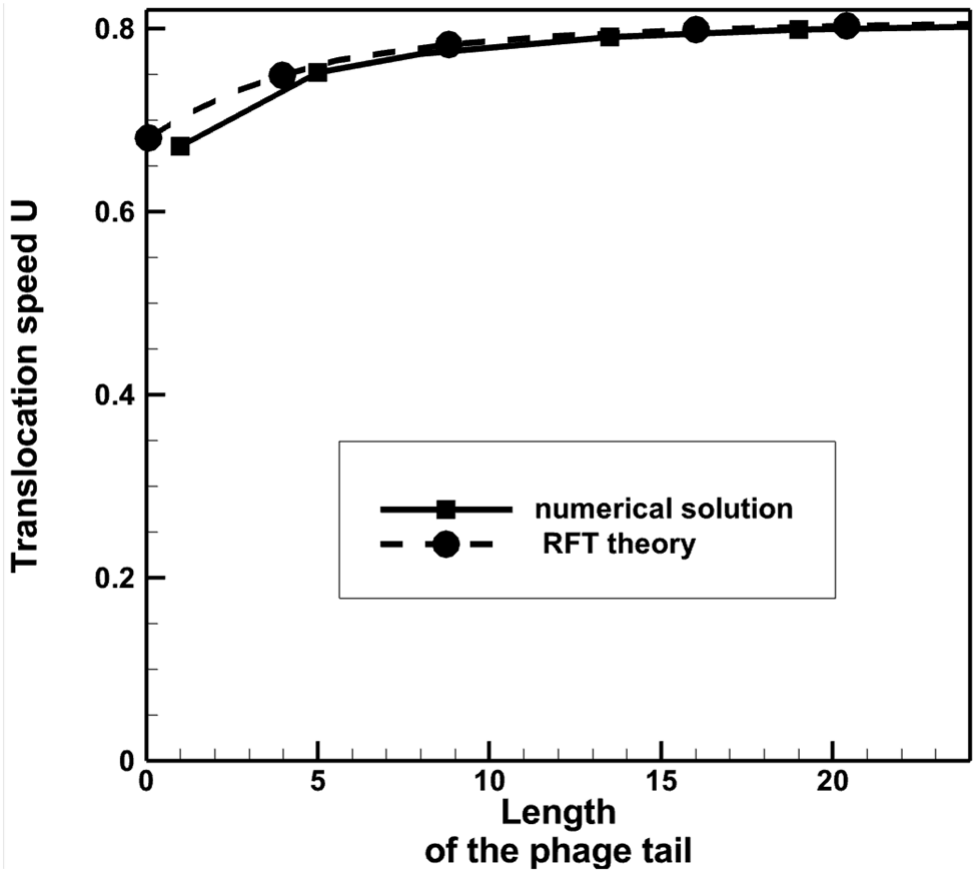
Dependency of the phage translocation speed on its tail length: the Stokes solution vs. the RFT theory from^7^ for model 2. Dimensionless units of velocity and length are used based on 6.28 μm/s and 10 nm, respectively.

**Figure 13:**
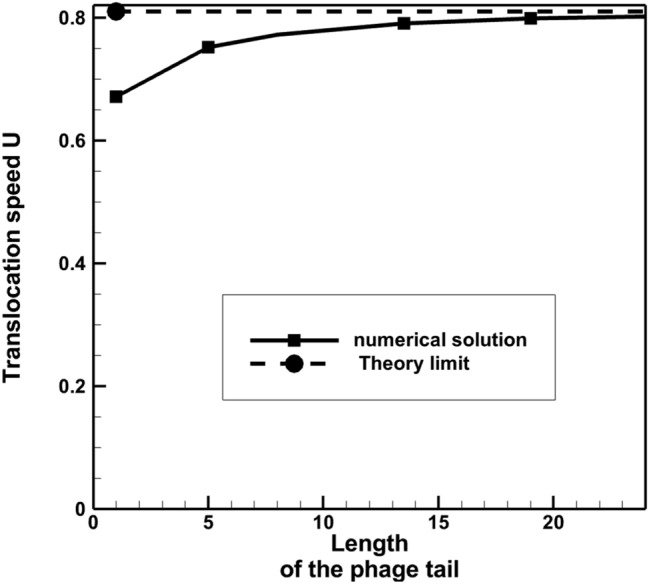
Dependency of the phage translocation speed on its tail length: comparison of the Stokes solution with the theoretical limiting case translocation speed applicable for very large phage tails. Dimensionless units of velocity and length are used based on 6.28 μm/s and 10 nm, respectively.

Figure 12 shows the comparison of the Stokes solution with the RFT solution for model 2. In this case both models predict very similar distributions of the translocation velocity, which increases with increasing the phage tail length, and contrary to the results of model 1. The good agreement of the RFT model with the Stokes solution here suggests that the guided motion of the phage fibre in the flagellum groove is reasonably well approximated by the analytical model, which assumes a fully developed shear flow resisting the sliding between the fiber and the surface of the groove. The longer the phage tail, the less rotation the phage has with respect to the ambient fluid, and the better the RFT approximaton holds. At long tail lengths the RFT solution and the Stokes solution virtually coincide. In Fig. 13, the Stokes solution of the translocation speed is compared with the theoretical limiting case solution, when the phage tail is so large that the phage stops rotating in the surrounding viscous fluid. In this case, the translocation speed of the phage is equal to the z-velocity component of the rotating helical groove of the flagellum, ${U=\omega }_{fl}{R}_{fl}\mathrm{cot}(\alpha )$. Notably, for the maximum tail length considered in the numerical simulations, the translocation speed is within 0.8% from the theoretical value.

The fibers immersed in the groove translocate 5–6 times faster compared to the smooth flagellum model 1 (Fig. 11), which is consitent with the previous discussion about the groove effect to efficiently translocate the phage without perturbing the viscous flow around the flagellum (Fig. 9).

Finally, the FEM solution of model 2 is examined in different scenarios by independently varying (a) the radius of the fibers r_fib_, (b) the size of the head a_h_, and (c) how deeply the fibers are within the groove by changing f_cov_, which is a parameter from^7^, standing for the circumference of the fiber cross section lying inside the groove. The three parameters are perturbed with respect to the baseline model configuration corresponding to r_fib_ = 1 nm, a_h_ = 30 nm, and f_cov_ = 0.5 at a phage length of 220 nm in accordance with the values in Table 1.

**Figure 14:**
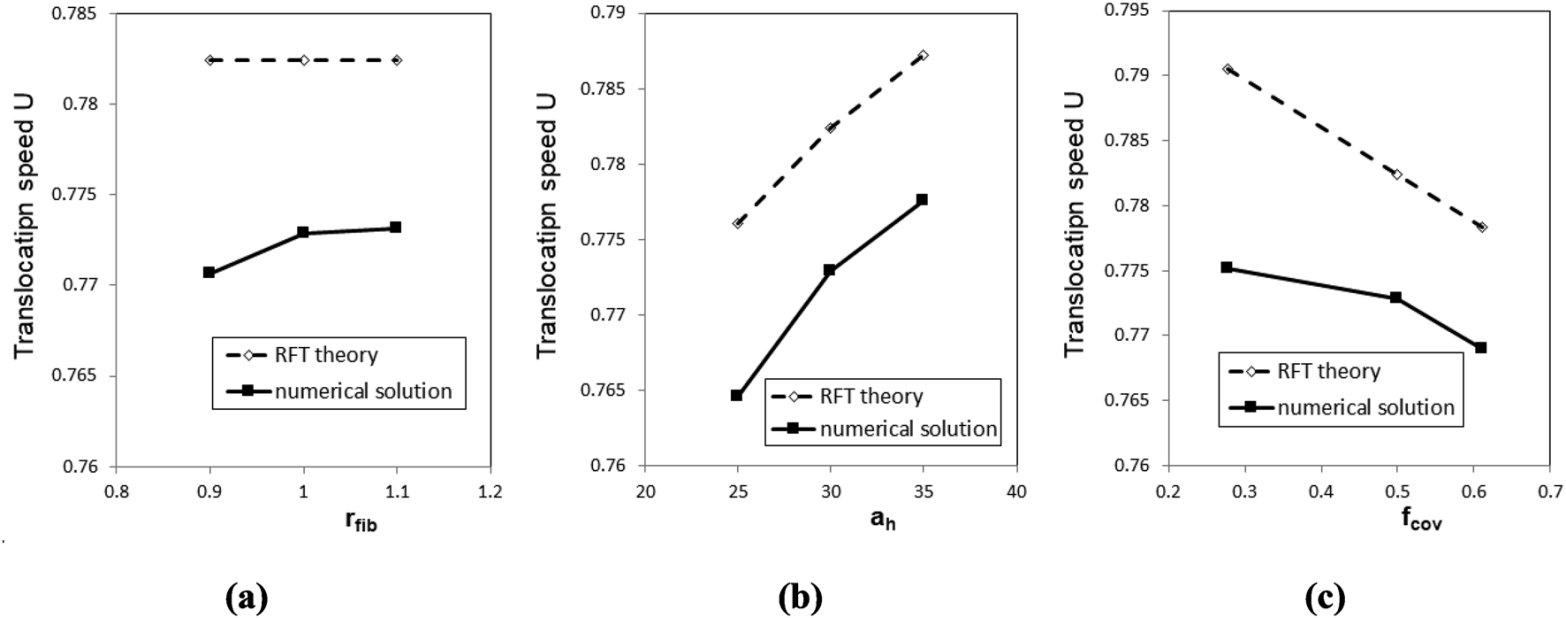
The dependence of the phage translocation speed on (**a**) the radius of the fibers r_fib_, (**b**) the size of the head a_h_, and (**c**) the circumference of the fiber cross section lying inside the groove f_cov_. Dimensionless units of velocity and length are used based on 6.28 μm/s and 1 nm, respectively.

The translocation speed predicted by FEM for scenarios (a), (b), and (c) is presented in Fig. 14a–c, respectively. In each case the solutions of the RFT model are included for comparison.

In comparison with the RFT solution the FEM predicts an increase of the translocation velocity with increasing the fiber radius for the same size of the groove (Fig. 14a). This discrepancy is likely due to ignoring 3D curvature effects of flagellum surface in the RFT model. The difference between the FEM and RFT solutions is more pronounced for a thinner fiber, which blocks less fluid volume inside the groove, thereby making the 3D flow effects including the outer flow entrainment more likely compared to a thicker fiber, which corresponds to a smaller gap with the groove wall.

The effect of increasing the phage head size is similar to increasing the phage tail length: a larger head leads to less rotation the phage has with respect to the ambient fluid (comp. Figs. 14b with 12). This trend is captured equally well by the FEM and the RFT models.

Figure 14c shows that a tighter location of the fibre inside the groove, which corresponds to a larger f_cov_, hence, larger friction between the fibers and the flagellum, leads to a smaller translocation speed in comparison to the scenario when a smaller part of the fiber is inside the groove. Both the FEM and the RFT models capture this trend, which is also consistent with a decay of the translocation speed with increasing the effective viscosity coefficient of the RFT model (see Fig. 9 in^7^). At the same time, a larger over-prediction of the translocation speed by the RFT model in comparison with the FEM solution is noted for smaller f_cov_. This discrepancy can be associated with a lesser shielding of the translocating fiber helix from the outer fluid by the groove for small f_cov_, thereby, a greater loss of the kinetic energy of the phage due to entraining parts of the viscous flow around the flagellum cylinder in comparison with the regime at large f_cov_, when fibers are more immersed in the groove. The latter effect is not expected to be resolved by the RFT model, which cannot capture a decrease of the translocation speed with reducing the fiber radius (Fig. 14a) or the effect of nonlocal reverse flow zones generated around the flagellum in the limiting case of zero f_cov_ (Fig. 9a).

## Discussion and conclusions

The ‘nut-and-bolt’ mechanism of a bacteriophage attacking a bacterium by harvesting the kinetic energy from its flagellum rotation, which was originally proposed in^8^ and modelled using Resistive Force Theory (RFT) in^7^, has been revisited using the fluid–structure-interaction model based on the Stokes equations. Following^19^, the linearised approach to represent the motion of the flagellum-phage complex in viscous inertialess fluid by a superposition of elementary translations and rotations of the phage and the flagellum is applied. For each elementary motion the Stokes equations are solved with non-slip boundary conditions of the 3D flagellum-phage geometry without any simplifying assumptions using a validated finite-element method. The numerical solution has been verified for a range of computational grids and computational domain sizes and shown to be non-sensitive to the numerical parameters. Notably, in comparison with the RFT model, the Stokes solution approach includes nonlocal hydrodynamic interactions and does not need any assumptions about the geometry-dependent friction coefficients. Following^7^, two mechanical models of the flagellum-phage complex have been considered. In the first model the phage fibre wraps around the smooth flagellum surface at some distance. In the second model the phage fibre is immersed in the flagellum surface which contains a helical groove mimicking the fibre shape. For both models the Stokes solution closely matches the trends previously reported using the RFT theory in^7^. The translocation speed of the phage decreases with the phage tail length in the first model and increases in the second model. The Stokes solution is in better agreement with the RFT solution for the second flagellum-phage model, which corresponds to a more tightly coupled configuration in comparison with the first model, which allows more slip. In line with the previous literature, it is also demonstrated that the predictions of the RFT method for the first model can be refined if the normal force friction coefficient is directly obtained from the Stokes solution to replace the analytical formula, which ignores the 3D curvature effects.

An important advantage of the FEM simulations compared to the RFT model is that these simulations can provide insights about the flow field in the entire fluid domain. Thus, the flow velocity distributions are computed in the vicinity of the flagellum for the scenario when the fibres are above the smooth flagellum surface and when the fibres are half-immersed in the groove. The comparison of the two velocity fields has revealed that in the second case the groove effectively shields the outer flow regions from the influence of the translocating helix, thereby providing a mechanism for the phage to translocate efficiently without notably perturbing the viscous flow around the flagellum.

For the grooved flagellum model a parameric investgation has been perfomed to separately analyse (i) the effect of the radius of the fibers, (ii) the size of the phage head, and (iii) how deeply the fibers are immersed within the groove. Increasing the phage head leads to reducing the translocation speed of the phage, in the same way as the previously reported effect of the phage tail length. The results of the FEM solution also show that reducing the radius of the fiber reduces the translocation speed of the phage, possibly, due to enlarging the gap beween the groove and the fibre. Furthermore, reducing the fraction of the fibre circumference immersed in the groove is shown to diminish the translation speed. While a reduction of the translocation speed with increasing the effective viscosity of the flow was reported for the RFT model before^7^, the suggested FEM model allows directly capturing the effect of increased friction between the fibre and the flagellum due to the geometry change in a more tightly coupled flagellum-phage configuration.

The suggested numerical approach based on solving the Stokes equations with a finite element method is flexibly extendable to any complex geometry and wall boundary conditions. When combined with detailed experimental measurements including video and continuous imaging, the current approach offers an attractive opportunity for modelling complex mechanical-flow interactions in bacterium-phage systems. In addition, future work will be devoted to extending the present Stokes flow method to a linearised Navier–Stokes formulation to incorporate potentially important unsteady effects in the modelling.

## Data Availability

The dataset used during the current study is available from the corresponding author upon reasonable request.
